# Age-Specific Strategies in Pediatric Vascular Trauma: A Comparative Analysis of Surgical and Heparin-Based Conservative Treatments

**DOI:** 10.7759/cureus.71823

**Published:** 2024-10-19

**Authors:** Nikolaos Giannakopoulos, Georgios Pitoulias, Sofia Tzamtzidou, Vangelis Bontinis, Dimitra Manou, Apostolos Pitoulias, Theofanis Papas

**Affiliations:** 1 Vascular Surgery, Korgialeneio-Benakeio Hellenic Red Cross Hospital, Athens, GRC; 2 Vascular Surgery, Second Department of Surgery, G. Gennimatas Hospital, Aristotle University of Thessaloniki, Thessaloniki, GRC; 3 Vascular Surgery, AHEPA University Hospital of Thessaloniki, Medical School, Aristotle University of Thessaloniki, Thessaloniki, GRC; 4 Vascular and Endovascular Surgery, Asklepios Clinic, Langen, DEU

**Keywords:** age-specific protocols, conservative treatment, heparin, limb salvage, pediatric vascular trauma, surgical intervention

## Abstract

Background

Pediatric vascular trauma, although rare, poses significant clinical challenges due to the potential for long-term morbidity, including limb compromise and growth abnormalities. This study addresses the gap in standardized treatment protocols by evaluating the efficacy of heparin-based conservative treatments compared to surgical interventions in children under 13 years of age.

Methods

A retrospective observational study was conducted at a referral center, reviewing institutional records from January 2010 to December 2020. The study included 27 pediatric patients aged up to 13 years who sustained arterial trauma involving the brachial and femoral arteries. Injuries were categorized as penetrating, blunt, or iatrogenic. Patients were divided into two age groups: those below six years and those six years and above. Treatment modalities were categorized into open surgical repair and medical management with unfractionated heparin. The diagnosis was confirmed through physical examination, Doppler ultrasound, and angiography.

Results

Out of 27 patients, 17 underwent surgical intervention, while 10 received medical management with heparin. An overall limb salvage rate of 87% was achieved. One case of limb loss occurred in a patient under six years who underwent surgical intervention. No significant limb-length discrepancies were observed. Surgical approaches, particularly interposition vein grafting, effectively restored palpable distal pulses. The results highlight the importance of an age-specific approach, demonstrating the effectiveness of both surgical and conservative treatments.

Conclusion

The study underscores the necessity for age-specific treatment protocols in pediatric vascular trauma. Individualized care resulted in high rates of limb salvage and favorable outcomes. These findings contribute to enhancing the understanding and management of pediatric vascular trauma, promoting the development of nuanced, age-tailored treatment protocols in clinical practice.

## Introduction

Pediatric vascular trauma, although uncommon, presents significant clinical challenges due to its potential for long-term morbidity, including limb compromise, growth abnormalities, and even amputation. The incidence of pediatric vascular injuries ranges from 0.6% to 1.4%, making them rare but clinically significant when they occur [1-3]. Upper extremity injuries, primarily resulting from penetrating trauma, account for approximately 37.9% of cases [3]. Lower extremity injuries, though less frequent at 20%, pose unique challenges due to the involvement of larger vessels and a greater likelihood of limb-threatening ischemia. Blunt trauma accounts for 29% of upper extremity injuries and 18% of lower extremity injuries, with the brachial and superficial femoral arteries (SFA) most commonly affected [3]. Involvement of these major vessels can lead to significant morbidity if not promptly addressed.

Management of these injuries in pediatric patients requires careful consideration of multiple factors. The smaller caliber of vessels in children, along with their unique physiological responses, can complicate surgical repair. This complexity is compounded by diagnostic and therapeutic uncertainties surrounding the use of anticoagulants and vascular reconstruction techniques in young patients. Studies have demonstrated the utility of unfractionated heparin in maintaining vascular patency and preventing thrombosis in pediatric patients [4-6], although the risk of complications, such as limb asymmetry following conservative treatment, remains a concern [5,7].

Current literature emphasizes the importance of early intervention to achieve high limb salvage rates, which can exceed 85% when managed appropriately [1,2]. However, the decision between conservative management using heparin and surgical intervention remains a point of debate. Surgical options, such as interposition vein grafting and end-to-end anastomosis, have shown promise in restoring blood flow and preventing limb loss, as evidenced by several studies [6,8]. Despite these advancements, the absence of standardized treatment protocols for pediatric patients, especially those under six years of age, leaves clinicians uncertain regarding the optimal course of treatment [3,9,10].

Furthermore, anatomical and physiological differences in younger children necessitate an age-specific approach to treatment [11,12]. In neonates and infants, smaller vessel size, greater risk of vascular spasm, and reduced collateral circulation can complicate both conservative and surgical interventions. These challenges are compounded by the potential for long-term complications such as limb-length discrepancies and delayed growth [13,14].

Iatrogenic injuries, which account for a notable proportion of pediatric vascular trauma cases, pose additional challenges. Studies highlight the frequency of these injuries, particularly following catheterizations or other invasive procedures, stressing the importance of careful technique and vigilant postoperative monitoring to prevent long-term sequelae [15,16]. These findings are corroborated by research advocating for a structured, algorithmic approach to managing vascular complications in pediatric patients, especially those resulting from iatrogenic causes [17].

Objectives

In light of the gaps in the literature, particularly regarding age-specific treatment protocols, our study aims to compare the efficacy of conservative heparin-based management with surgical intervention in pediatric patients under the age of 13 who have sustained vascular injuries to the upper or lower extremities. We specifically aim to evaluate the outcomes in two distinct age groups, children under six years of age and those aged six years and above, to determine whether age-specific treatment strategies are warranted. The primary outcome of this study is the limb salvage rate, defined as the successful preservation of the injured limb without the need for amputation. Secondary outcomes include the incidence of limb-length discrepancies (limb asymmetry), necessity for amputation, restoration of palpable distal pulses post-procedure, and any treatment-related complications. By assessing both early and long-term outcomes across different age groups, we hope to contribute to the development of standardized, age-specific treatment protocols that account for the unique physiological characteristics of pediatric patients.

## Materials and methods

A retrospective observational study was conducted over a ten-year period from January 2010 to December 2020 at Korgialeneio-Benakeio Hellenic Red Cross Hospital, a tertiary care center in Athens, Greece. Institutional hospital records were reviewed to identify pediatric patients up to 13 years of age who sustained arterial trauma to the upper or lower extremities. The study included patients with injuries classified as penetrating, blunt, or iatrogenic, provided that complete medical records and follow-up data were available. Patients with pre-existing vascular conditions or incomplete medical records were excluded from the study.

Ethical considerations

The study was conducted in accordance with the principles outlined in the Declaration of Helsinki. Ethical approval was obtained from the Institutional Review Board (IRB) of Korgialeneio-Benakeio Hellenic Red Cross Hospital. Due to the retrospective nature of the study and the use of anonymized patient data, the requirement for informed consent was waived by the IRB.

Two age groups were defined based on developmental considerations and clinical relevance: Group A consisted of children below six years of age, and Group B included children aged six years and above. This age cutoff was chosen to reflect significant anatomical and physiological differences that influence treatment decisions and outcomes in pediatric vascular trauma. By categorizing patients into these two groups, we aimed to compare the efficacy of conservative heparin-based management and surgical interventions within each age group to assess the necessity for age-specific treatment strategies.

Children under six years of age have smaller vessel calibers, increased vessel fragility, and a higher propensity for vasospasm, which can complicate surgical interventions and increase the risk of thrombosis. Their hemodynamic parameters and responses to anticoagulation differ significantly from older children, affecting the dosing and monitoring of medications like heparin. Additionally, the development of collateral circulation is less mature in this age group, potentially impacting the success of both conservative and surgical treatments.

In contrast, children aged six years and above have more developed vascular systems with larger vessel calibers and more robust collateral circulation. Their physiological responses are closer to those of adults, allowing for a broader range of therapeutic options and more predictable outcomes. This categorization allowed for a more precise assessment of age-specific treatment strategies and their effectiveness [11,13].

Diagnosis and treatment decision criteria

The diagnosis was based on clinical assessment, including evaluation of pulses, capillary refill, limb perfusion, and signs of ischemia, and was confirmed with Doppler ultrasound and angiography when necessary. Treatment decisions were guided by injury severity, presence of palpable pulses, Doppler ultrasound findings, and hemodynamic stability.

Medical management

Medical management was indicated for patients with positive distal Doppler signals, palpable pulses, and stable hemodynamics. These patients received unfractionated heparin, administered as an initial bolus of 100 units per kilogram intravenously, followed by a continuous infusion of 20 to 25 units per kilogram per hour. Activated partial thromboplastin time (aPTT) was monitored every six hours to adjust dosing and maintain therapeutic levels.

Surgical intervention

Surgical intervention was indicated for patients with absent pulses, significant arterial damage evident on imaging or exploration, or life-threatening bleeding. Surgical techniques employed included interposition vein grafting, end-to-end anastomosis, patch angioplasty, and bypass procedures, tailored to the specific anatomical and injury characteristics of each case. Perioperative anticoagulation involved an intraoperative bolus of unfractionated heparin at 50 units per kilogram prior to vascular clamping. Postoperatively, low-molecular-weight heparin (enoxaparin) was administered at 1 milligram per kilogram every 12 hours for five to seven days to prevent thrombosis. All surgical procedures were performed by a board-certified vascular surgeon with pediatric expertise.

Data collection

Data extracted from medical records included patient demographics (age and sex), injury characteristics (mechanism: penetrating, blunt, or iatrogenic, and location: brachial artery, superficial femoral artery, or popliteal artery), treatment modalities (medical management or surgical intervention), and outcomes. Outcomes assessed were limb salvage (primary outcome), presence of palpable pulses post-procedure, limb-length discrepancies, the necessity for amputation, and any treatment-related complications (secondary outcomes). Additionally, outcomes were compared between the two age groups to evaluate the effectiveness of treatment modalities within each group and to determine if age-specific strategies are beneficial.

Statistical analysis

Statistical analyses were performed using R statistical software version 4.0.3 (R Foundation for Statistical Computing, Vienna, Austria). Data manipulation and handling were conducted using the tidyverse package (version 1.3.0). For creating graphs and visualizations, the ggplot2 package (version 3.3.2) was utilized. The reshape2 package (version 1.4.4) was employed for data reshaping necessary for certain analyses. Statistical tests, including chi-square tests and Fisher's exact tests, were performed using the stats base R package.

Categorical variables were compared using the chi-square test when the expected frequencies were adequate, and Fisher's exact test was applied when the expected frequencies were less than five in any of the contingency table cells. A p-value of less than 0.05 was considered statistically significant. Graphs and figures were generated using ggplot2 to visually represent the data and support statistical findings. The types of graphs created included bar charts, heatmaps, pie charts, stacked bar charts, and box plots to effectively illustrate the study's results.

## Results

Patient demographics and treatment modalities

**Figure 1:**
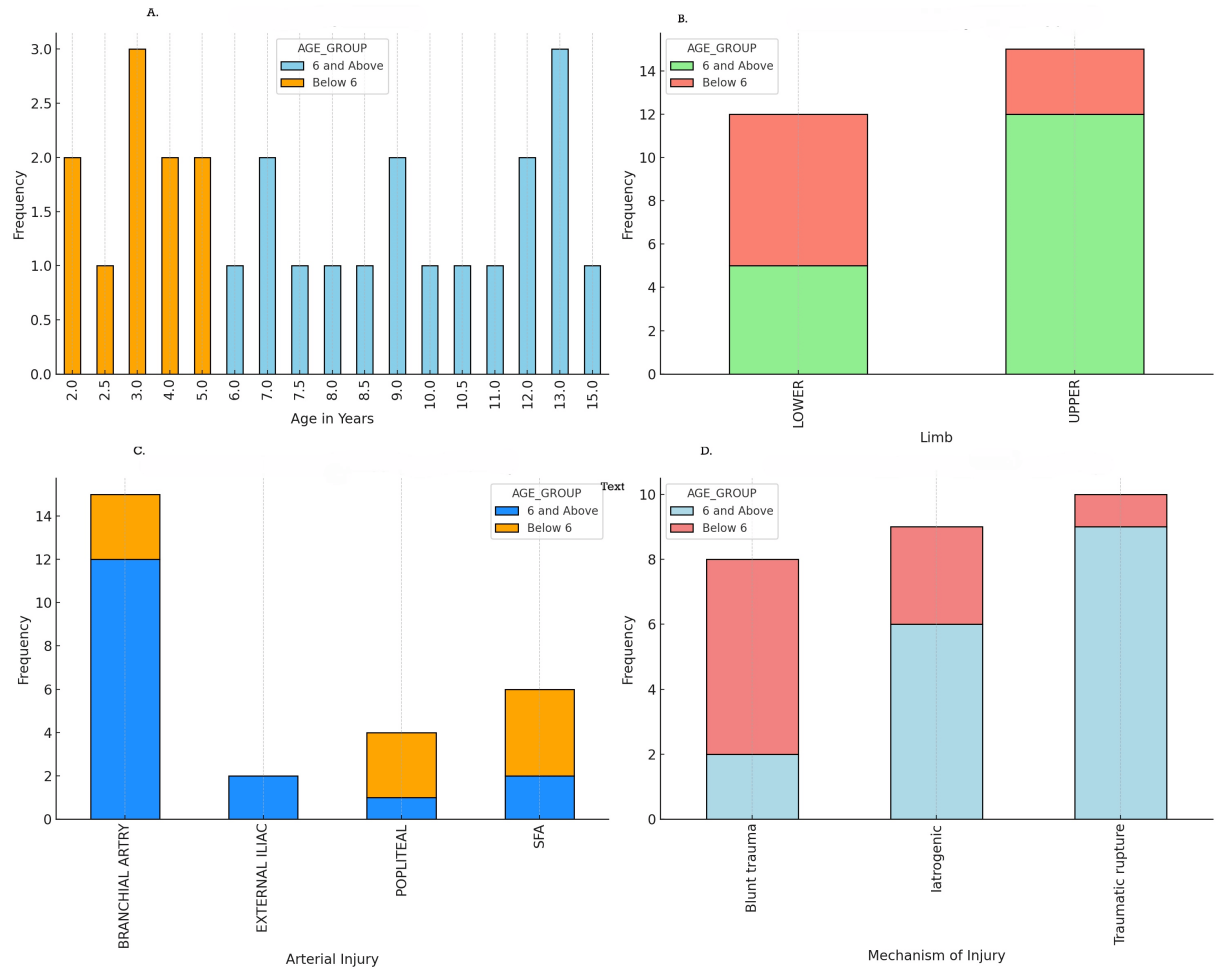
Distribution of patients among different treatment modalities and surgical techniques. A: age distribution of patients; B: limb affected by age group; C: types of arterial injuries by age group; D: mechanism of injury by age group

Our study analyzed 27 pediatric patients, categorized into two age groups. This categorization allowed us to precisely assess and compare the outcomes of treatments within these age-specific groups: six years and above (n = 17, 63%) and below six years (n = 10, 37%). The majority of injuries were to the upper limbs, with 15 patients (55.6%) sustaining brachial artery injuries. Lower limb injuries accounted for 12 patients (44.4%), predominantly involving the superficial femoral artery (SFA) in six patients (22.2%) and the popliteal artery in four patients (14.8%). Two patients (7.4%) had injuries to the external iliac artery. Seventeen patients (63%) underwent surgical intervention, while 10 (37%) received medical management using unfractionated heparin. Among the surgical interventions, interposition vein grafting was the most common technique, performed in 11 patients (64.7% of surgical cases). End-to-end anastomosis was conducted in four patients (23.5%), and bypass procedures and patch angioplasty were performed in two patients (11.8%) (Appendix 1). The distribution of patients among different treatment modalities and surgical techniques is detailed in Figure 1.

Patient outcomes and treatment efficacy

**Figure 2:**
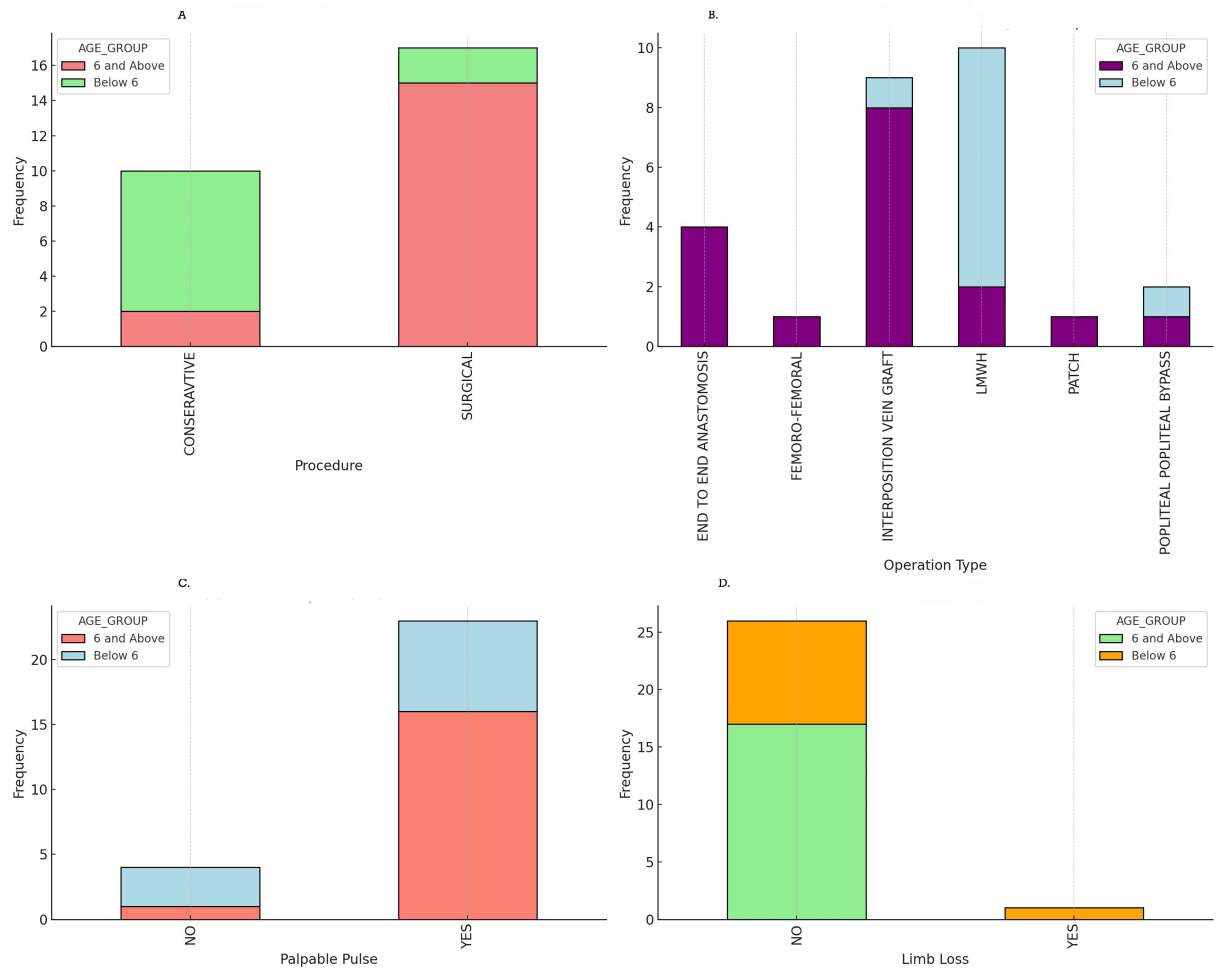
Detailed outcome. A: types of procedures by age group; B: operation types by age group; C: post-procedure palpable pulse by age group; D: limb loss by age group

**Figure 3:**
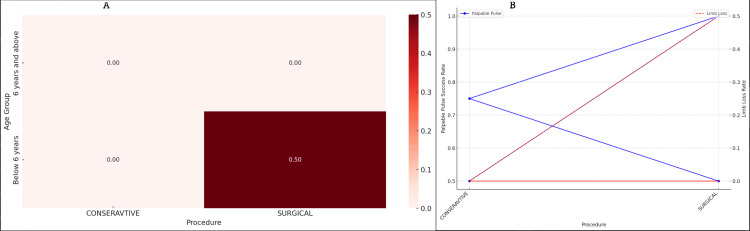
Patient outcome. A: heatmap of limb loss rates; B: palpable pulse and limb loss success rates by procedure type

The overall limb salvage rate in our cohort was high at 96.3% (26 out of 27 patients). Limb loss occurred in only one patient (3.7%), a child under six years of age who underwent surgical intervention, highlighting the potential risks associated with surgical treatments in this age group. No limb-length discrepancies were observed post-treatment, indicating the effectiveness of both surgical and medical management in preserving limb integrity. Restoration of palpable distal pulses post-procedure was achieved in 23 out of 27 patients (85.2%). In patients below six years, palpable pulses were restored in seven out of 10 patients (70%), whereas in patients aged six years and above, palpable pulses were restored in 16 out of 17 patients (94.1%). The difference in palpable pulse rates between the age groups was not statistically significant (p = 0.105), suggesting a potential trend that may warrant further investigation with a larger sample size. Regarding limb loss rates, in the group of patients below six years old, limb loss occurred in one out of 10 patients (10%), while no limb loss was observed in patients aged six years and above (zero out of 17 patients (0%)). The difference in limb loss rates between the age groups was not statistically significant (p = 0.333). However, among the surgical patients under six years old, limb loss occurred in one out of two patients (50%), compared to no limb loss in the surgical patients aged six years and above (zero out of 15 patients (0%)). This difference approached statistical significance (p = 0.0667), highlighting a potential increased risk associated with surgical interventions in the younger age group. Detailed outcomes for each age group and treatment modality are summarized in Figures 2, 3.

Surgical techniques and involved arteries

Among the 17 patients who underwent surgical intervention, various techniques were employed based on the specific needs and anatomical considerations of each case. The most common surgical method was interposition vein grafting, performed in nine patients, which accounts for 52.9% of the surgical cases. All these procedures involved the brachial artery, highlighting its prominence in upper limb vascular injuries. Another frequently utilized technique was end-to-end anastomosis, carried out in four patients (23.5% of surgical cases), also involving the brachial artery. This method was chosen when the arterial ends could be approximated without tension, typically in less extensive injuries. In addition to these, popliteal-popliteal bypass surgeries were performed in two patients (11.8% of surgical cases) who had injuries to the popliteal artery. This complex procedure was necessary due to the location and severity of the arterial damage. Femoro-femoral bypass and patch angioplasty were each performed in one patient (5.9%, respectively), both addressing injuries to the superficial femoral artery (SFA). These less common procedures were selected based on specific injury patterns and the surgeons' assessment of the most effective method to restore arterial flow. The arteries involved in these surgical interventions predominantly included the brachial artery, accounting for 76.5% (13 out of 17) of the surgical cases. This high percentage underscores the susceptibility of the brachial artery to traumatic injuries in pediatric patients, possibly due to its superficial location and frequent exposure during activities common in this age group. The popliteal and superficial femoral arteries were each involved in 11.8% of the surgical cases (two patients each), reflecting the occurrence of lower limb arterial injuries that necessitate surgical repair. The outcomes associated with these surgical techniques were notably positive. Restoration of palpable distal pulses post-procedure was achieved in 94.1% (16 out of 17) of the surgical patients, indicating a high success rate in re-establishing adequate blood flow to the affected limbs. Specifically, all patients who underwent interposition vein grafting and end-to-end anastomosis experienced successful restoration of palpable pulses, demonstrating the efficacy of these techniques in brachial artery repairs. However, one case resulted in limb loss, accounting for 5.9% of the surgical patients. This patient was under six years old and had undergone a popliteal-popliteal bypass due to a severe injury to the popliteal artery. The complexity of the procedure, combined with the patient's young age and the challenges associated with smaller vessel size and increased risk of thrombosis, likely contributed to the unfavorable outcome. This incident highlights the increased risks associated with surgical interventions in younger children, particularly for complex lower limb arterial injuries. A complete breakdown of surgical techniques and the arteries involved is presented in Figure 2.

## Discussion

Our investigation into pediatric arterial trauma management has revealed significant insights, specifically emphasizing the importance of age-adapted treatment protocols and the balance between surgical and conservative approaches. With an overall limb salvage rate of 96.3%, our findings align with other notable studies, such as those by Corneille et al. [1] and Wang et al. [2], which reported high rates of successful limb salvage when early intervention was utilized. However, our findings further highlight age-specific factors that play a crucial role in determining treatment outcomes in pediatric patients.

Age remains a pivotal factor in managing pediatric vascular trauma. Our analysis focused heavily on children below the age of six, a demographic frequently overlooked in trauma research, as noted by LaQuaglia et al. [13] and Friedman et al. [11]. These younger patients face unique challenges due to smaller vessel caliber, increased risk of vasospasm, and limited physiological reserve, making conservative approaches such as heparin-based therapy potentially more appropriate. In our cohort, younger patients had a higher rate of surgical complications, including one instance of limb loss, reinforcing the notion that non-surgical management should be strongly considered in select cases.

In contrast, older pediatric patients, particularly those aged six years and above, benefited more from surgical interventions. Surgical techniques such as interposition vein grafting, which was the most common in our cohort, were associated with a high rate of palpable pulse restoration and limb salvage. The success of these procedures is consistent with findings by Alves et al. [6] and Sciarretta et al. [15], who demonstrated that surgical techniques are more effective in older children with well-established vasculature. Moreover, the use of perioperative heparin and postoperative low-molecular-weight heparin (LMWH) for anticoagulation in our study proved essential for preventing thrombosis and ensuring graft patency, mirroring protocols recommended by Chaikof et al. [5] and Taylor et al. [18].

Timely diagnosis and intervention are critical factors influencing outcomes in pediatric vascular trauma. Delays can lead to prolonged ischemia, increasing the risk of limb loss and long-term functional impairment. In our study, prompt assessment using clinical evaluation and imaging modalities such as Doppler ultrasound and angiography facilitated early treatment decisions. Emphasizing the importance of early recognition and intervention aligns with the findings of Wang et al. [2], who reported improved outcomes with expedited care.

Our study found that children under six years responded well to conservative management with unfractionated heparin. This finding challenges the surgical bias prevalent in pediatric vascular trauma, particularly in younger children. Heparin-based management helped prevent the need for more invasive surgery while effectively maintaining limb perfusion in the majority of cases. These results are supported by the work of Markovic et al. [4], who also documented the effectiveness of conservative treatment in preventing limb loss in pediatric patients.

However, it is crucial to balance the benefits of heparin-based management with the risks of anticoagulation, particularly in very young children. As Chaikof et al. [5] and Taylor et al. [18] noted, complications such as bleeding and limb asymmetry can arise from heparin use, necessitating careful patient selection and diligent monitoring. In our cohort, no significant bleeding complications were noted, but one case of limb asymmetry was observed, which underscores the delicate nature of conservative management in this patient population.

For older children, surgical repair techniques such as interposition vein grafting, patch reconstruction, and bypasses were effective in restoring limb function and achieving long-term patency. The successful use of interposition vein grafts, particularly in the upper extremities, was consistent with reports by Alves et al. [6], who documented excellent functional outcomes following such procedures. Additionally, Dalsing et al. [7] and Lin et al. [17] demonstrated that older children tend to have fewer complications post-surgery and better long-term outcomes, with most patients maintaining limb length and function.

Our study did not observe significant limb-length discrepancies post-surgery, which echoes the findings of Sciarretta et al. [15] and Wahlgren et al. [16] concerning the success of contemporary surgical techniques in preserving limb integrity and function. However, as LaQuaglia et al. [13] noted, there remains a risk of long-term growth-related complications in younger patients, especially when surgery is performed at an early age. This reinforces the need for long-term follow-up to monitor growth patterns and ensure limb symmetry in pediatric trauma patients.

The distribution of injury mechanisms in our cohort, which included both blunt trauma and iatrogenic injuries, reflects the findings of Barmparas et al. [3] and Sciarretta et al. [15], who identified a similar distribution in pediatric vascular trauma. Blunt trauma, particularly in the lower extremities, often necessitates more aggressive interventions due to the risk of ischemia and nerve injury, while iatrogenic injuries are more likely to occur in the upper extremities during medical procedures. In our study, upper extremity injuries, particularly brachial artery injuries, were more prevalent and were associated with better outcomes, likely due to the ease of surgical access and availability of suitable autologous grafts, as supported by the work of Alves et al. [6] and Dalsing et al. [7].

Despite the valuable insights gained from this study, it is not without limitations. The retrospective nature of our analysis, combined with the relatively small sample size, limits the generalizability of our findings. As noted in the work of Wang et al. [2] and Lin et al. [17], larger prospective studies are necessary to further validate the efficacy of age-specific protocols and refine treatment algorithms for pediatric vascular trauma. Additionally, the lack of neonates in our cohort may have introduced referral bias, as younger patients with severe trauma may have been referred to specialized pediatric centers. Future research should include a broader range of ages, including neonates, to fully understand the long-term impact of different treatment modalities. Moreover, studies focusing on the psychosocial impact and quality of life of pediatric trauma survivors are needed to complement the existing body of literature.

## Conclusions

In conclusion, our study emphasizes the critical role of age-specific strategies in the management of pediatric vascular trauma. By including age as a significant factor in our objectives and methodology, we aimed to determine the most effective treatment modalities for different pediatric age groups. Our findings indicate that while surgical interventions are effective for older children, conservative heparin-based management shows promise in younger children, particularly those under six years of age. The choice of treatment must be individualized, taking into account the patient's age, mechanism of injury, and potential risks associated with each approach. By advocating for multidisciplinary care, integrating technological advancements, and emphasizing the need for standardized protocols and long-term follow-up, we aim to contribute to improved patient outcomes and long-term limb function in pediatric vascular trauma patients. Our findings contribute to a growing body of literature advocating for tailored, age-specific treatment protocols in pediatric vascular trauma, paving the way for enhanced clinical practice and patient care.

## Appendices

Appendix 1 

**Table 1:** Dataset details. ID: patient identifier; sex: gender of the patient; age in years: age of the patient; limb: whether the injury was in the upper or lower limb; pre-procedure Doppler signs: presence of Doppler signals before the procedure (yes/no); papable pulse: presence of a palpable pulse before the procedure (yes/no); arterial injury: type of arterial injury (e.g., brachial artery); procedure: type of procedure (conservative/surgical); operation type: type of surgical operation performed; post-procedure Doppler signs: presence of Doppler signals after the procedure (yes/no); post-procedure palpable pulse: presence of palpable pulse after the procedure (yes/no); mechanism of injury: cause of injury (e.g., iatrogenic, trauma); limp loss: limb loss as an outcome (yes/no); limb length discrepancies: limb length discrepancies as an outcome (yes/no) LWMH: low-molecular-weight heparin

ID	Sex	Age in years	Limb	Pre-procedure Doppler signs	Palpable pulse	Arterial injury	Procedure	Operation type	Post-procedure Doppler signs	Post-procedure palpable pulse	Mechanism of injury	Limp loss	Limb length discrepancies
1	M	8	Upper	No	No	Brachial artery	Surgical	End-to-end anastomosis	Yes	Yes	Iatrogenic	No	No
2	M	10	Upper	No	No	Brachial artery	Surgical	End-to-end anastomosis	Yes	Yes	Iatrogenic	No	No
3	M	7	Upper	No	No	Brachial artery	Surgical	End-to-end anastomosis	Yes	Yes	Iatrogenic	No	No
4	M	13	Upper	No	No	Brachial artery	Surgical	End-to-end anastomosis	Yes	Yes	Iatrogenic	No	No
5	M	6	Upper	No	No	Brachial artery	Surgical	Interposition vein graft	Yes	Yes	Iatrogenic	No	No
6	F	8.5	Upper	No	No	Brachial artery	Surgical	Interposition vein graft	Yes	Yes	Iatrogenic	No	No
7	F	2	Upper	No	No	Brachial artery	Surgical	Interposition vein graft	Yes	Yes	Traumatic rupture	No	No
8	F	12	Upper	No	No	Brachial artery	Surgical	Interposition vein graft	Yes	Yes	Traumatic rupture	No	No
9	M	7	Upper	No	No	Brachial artery	Surgical	Interposition vein graft	Yes	Yes	Traumatic rupture	No	No
10	M	7.5	Upper	No	No	Brachial artery	Surgical	Interposition vein graft	Yes	Yes	Traumatic rupture	No	No
11	F	9	Upper	No	No	Brachial artery	Surgical	Interposition vein graft	Yes	Yes	Traumatic rupture	No	No
12	M	10.5	Upper	No	No	Brachial artery	Surgical	Interposition vein graft	Yes	Yes	Traumatic rupture	No	No
13	F	13	Upper	No	No	Brachial artery	Surgical	Interposition vein graft	Yes	Yes	Traumatic rupture	No	No
14	F	13	Lower	No	No	SFA	Surgical	Femoro-femoral	Yes	Yes	Traumatic rupture	No	No
15	F	15	Lower	No	No	SFA	Surgical	Patch	Yes	Yes	Traumatic rupture	No	No
16	M	12	Lower	No	No	Popliteal	Surgical	Popliteal popliteal bypass	Yes	Yes	Traumatic rupture	No	No
17	M	2.5	Lower	No	No	Popliteal	Surgical	Popliteal popliteal bypass	No	No	Iatrogenic	Yes	No
18	M	9	Lower	Yes	No	External iliac	Conservative	LMWH	Yes	Yes	Blunt trauma	No	No
19	M	11	Lower	Yes	No	External iliac	Conservative	LMWH	Yes	No	Blunt trauma	No	No
20	M	3	Upper	Yes	No	Brachial artery	Conservative	LMWH	Yes	No	Blunt trauma	No	No
21	M	2	Upper	Yes	No	Brachial artery	Conservative	LMWH	Yes	Yes	Blunt trauma	No	No
22	M	4	Lower	Yes	No	SFA	Conservative	LMWH	Yes	Yes	Blunt trauma	No	No
23	M	5	Lower	Yes	No	SFA	Conservative	LMWH	Yes	Yes	Blunt trauma	No	No
24	M	3	Lower	Yes	No	Popliteal	Conservative	LMWH	Yes	Yes	Blunt trauma	No	No
25	M	4	Lower	Yes	No	SFA	Conservative	LMWH	Yes	No	Blunt trauma	No	No
26	M	3	Lower	Yes	No	Popliteal	Conservative	LMWH	Yes	Yes	Iatrogenic	No	No
27	M	5	Lower	Yes	No	SFA	Conservative	LMWH	Yes	Yes	Iatrogenic	No	No

## References

[1] Corneille MG, Gallup TM, Villa C (2011). Pediatric vascular injuries: acute management and early outcomes. J Trauma.

[2] Wang SK, Drucker NA, Raymond JL (2019). Long-term outcomes after pediatric peripheral revascularization secondary to trauma at an urban level I center. J Vasc Surg.

[3] Barmparas G, Inaba K, Talving P (2010). Pediatric vs adult vascular trauma: a National Trauma Databank review. J Pediatr Surg.

[4] Markovic MD, Cvetkovic SD, Koncar IB (2019). Treatment of pediatric vascular injuries: the experience of a single non-pediatric referral center. Int Angiol.

[5] Chaikof EL, Dodson TF, Salam AA, Lumsden AB, Smith RB 3rd (1992). Acute arterial thrombosis in the very young. J Vasc Surg.

[6] Alves K, Spencer H, Barnewolt CE, Waters PM, Bae DS (2018). Early outcomes of vein grafting for reconstruction of brachial arterial injuries in children. J Hand Surg Am.

[7] Dalsing MC, Cikrit DF, Sawchuk AP (2005). Open surgical repair of children less than 13 years old with lower extremity vascular injury. J Vasc Surg.

[8] Morão S, Ferreira RS, Camacho N (2018). Vascular trauma in children-review from a major Paediatric Center. Ann Vasc Surg.

[9] Downey C, Aliu O, Nemir S, Naik-Mathuria B, Hatef DA, Bullocks JM, Friedman JD (2013). An algorithmic approach to the management of limb ischemia in infants and young children. Plast Reconstr Surg.

[10] Dua A, Via KC, Kreishman P (2013). Early management of pediatric vascular injuries through humanitarian surgical care during U.S. military operations. J Vasc Surg.

[11] Friedman J, Fabre J, Netscher D, Jaksic T (1999). Treatment of acute neonatal vascular injuries--the utility of multiple interventions. J Pediatr Surg.

[12] Kumar R, Trikha V, Malhotra R (2001). A study of vascular injuries in pediatric supracondylar humeral fractures. J Orthop Surg (Hong Kong).

[13] LaQuaglia MP, Upton J, May JW Jr (1991). Microvascular reconstruction of major arteries in neonates and small children. J Pediatr Surg.

[14] Lewis HG, Morrison CM, Kennedy PT, Herbert KJ (2003). Arterial reconstruction using the basilic vein from the zone of injury in pediatric supracondylar humeral fractures: a clinical and radiological series. Plast Reconstr Surg.

[15] Sciarretta JD, Macedo FI, Chung EL, Otero CA, Pizano LR, Namias N (2014). Management of lower extremity vascular injuries in pediatric trauma patients: a single Level I trauma center experience. J Trauma Acute Care Surg.

[16] Wahlgren CM, Kragsterman B (2015). Management and outcome of pediatric vascular injuries. J Trauma Acute Care Surg.

[17] Lin PH, Dodson TF, Bush RL (2001). Surgical intervention for complications caused by femoral artery catheterization in pediatric patients. J Vasc Surg.

[18] Taylor LM Jr, Troutman R, Feliciano P, Menashe V, Sunderland C, Porter JM (1990). Late complications after femoral artery catheterization in children less than five years of age. J Vasc Surg.

